# Dataset of biological community structure in Deepor Beel using eDNA approach–A RAMSAR wetland of Assam, India

**DOI:** 10.1016/j.dib.2023.109786

**Published:** 2023-11-07

**Authors:** Rajkumari Nikita, Anwesha Ghosh, Yash, Chakresh Kumar, Arkaprava Mandal, Nirupama Saini, Sourabh Kumar Dubey, Kalpajit Gogoi, Francois Rajts, Ben Belton, Punyasloke Bhadury

**Affiliations:** a Centre for Climate and Environmental Studies, Indian Institute of Science Education and Research Kolkata, Mohanpur-741246, Nadia, West Bengal, India; b Integrative Taxonomy and Microbial Ecology Research Group, Department of Biological Sciences, Indian Institute of Science Education and Research Kolkata, Mohanpur- 741246, Nadia, West Bengal, India; c WorldFish, Guwahati-781022, Assam, India; d Department of Agricultural, Food, and Resource Economics, Michigan State University, East Lansing, MI 48824, USA; e International Food Policy Research Institute, Gulshan 2, Dhaka 1212, Bangladesh; f Faculty of Applied Sciences, UCSI University Kuala Lumpur, UCSI Heights, Cheras, Kuala Lumpur 56000, Malaysia

**Keywords:** Freshwater, Nanopore, Bacterioplankton, Chordata

## Abstract

Deepor Beel, located in the state of Assam in India, is a Wetland of International Importance with a Wildlife Sanctuary and is the only RAMSAR site in the state. Though of invaluable ecological significance, the wetland is facing anthropogenic stressors, leading to rapid degradation of ecological health. In December 2022, surface water was collected from six stations of Deepor Beel to elucidate biological communities using the eDNA approach. At the time of sampling, *in-situ* environmental parameters were measured in triplicates. The dissolved nutrients and concentrations of metals and metalloids were estimated using UV–Vis Spectrophotometry and ICP-MS approaches respectively. The study revealed a high concentration of dissolved nitrate in the surface water. High-throughput sequencing using Nanopore sequencing chemistry in a MinION platform indicated the overwhelming abundance of Moraxellaceae (Prokaryotes) and Eumetazoa (Eukaryotes). The abundance of Cyprinidae were also encountered in the studied wetland reflecting the biodiversity of fish populations. High nitrate along with elucidated microbial signals are crucial to designate ecological health status of Deeper Beel. This study is aimed at generating baseline information to aid long-term monitoring and restoration of the Deepor Beel as well as the first comprehensive assessment of a RAMSAR Site located in northeast of India.

Specifications Table

**Table:** 

Subject	Environmental Science
Specific subject area	Microbial Ecology
Data format	Raw and analyzed
Type of data	Figures and Tables
How data were acquired	Field sampling using hand-held instruments, ICP-MS, environmental DNA extraction, Nanopore MinION MG-RAST, MEGAN7, RStudio 2023.03.1 + 446, ggplot2
Data collection	*In-situ* environmental parameters were measured in triplicates during sampling using handheld probes with ATC configurations. Surface water samples were collected in 1 L HDPE wide-mouth amber bottles and fixed with buffered 4 % formalin for dissolved nutrients estimation. Surface water samples were collected using 1 L wide-mouth white HDPE bottles. Collected samples were immediately fixed with molecular grade absolute ethanol for environmental DNA extraction (eDNA) and subsequent elucidation of microbial community structure.
Data source location	City and Town: Guwahati Region: Assam Country: India Latitude and Longitude: DPB1 (26.114 N 91.661E) DPB2 (26.117 N 91.663E) DPB3 (26.119 N 91.661E) DPB4 (26.117 N 91.658E) DPB5 (26.114 N 91.659E) DPB6 (26.113 N 91.659E)
Data accessibility	Repository name: SRA of NCBI Data identification number: SAMN35820160, SAMN35820161 Direct URL to data: https://www.ncbi.nlm.nih.gov/sra/PRJNA986129

## 1 Value of the Data

• The generated dataset provides baseline information including proxies of anthropogenic forcings such as high concentrations of dissolved nitrate that can help towards tracking changing ecological health of Deepor Beel
• The generated eDNA dataset which is the first for any RAMSAR wetland located in the northeast of India can act as a biological proxy towards assessing ecological health including impacts of pollutants on biodiversity
• The dataset will help the scientific community, policymakers and ecosystem managers engaged in wetland management and for targeted ecological restoration
• Further information added to the reported datasets can help track the effects of anthropogenic forcings on the functioning of microbial communities and resulting changes on the overall biodiversity of Deepor Beel wetland.

## 2 Data Description

The datasets described in this article are of prokaryotic and eukaryotic communities which were elucidated from eDNA extracted from surface water representing two stations of Deepor Beel, a RAMSAR wetland and Wildlife Sanctuary, located in the southwest corner of Guwahati City, Assam, India. Besides, *in*-*situ* environmental data, concentration of dissolved nutrients, metals and metalloids have been also deduced. The first station, DPB1, is inundated with an invasive aquatic plant species, the water hyacinth (*Pontederia crassipes*) and also receives flow of untreated effluents as well as dumping of mixed solid waste. On the other hand, the other selected station, DPB3, is located north of the wetland and there is no visible sign of human induced disturbances. Measured environmental parameters are shown in Table 1. The concentration of metals and metalloids present in the surface water of DPB1, DPB3 and DPB5 are shown in Table 2.

**Table 1:** Environmental parameters measured from surface water collected from six stations of Deepor Beel, Assam, India.

Environmental Parameters	DPB1	DPB2	DPB3	DPB4	DPB5	DPB6
AT ( °C)	20.1 ± 0	19.5 ± 0	21.6 ± 0	21.7 ± 0	21.7 ± 0	27.8 ± 0
SWT ( °C)	20.1 ± 0	19.5 ± 0	21.6 ± 0	21.7 ± 0	21.7 ± 0	25 ± 0
DO (mg/L)	6.7 ± 0	5.89 ± 0	5.62 ± 0	6.5 ± 0	6.2 ± 0	7.8 ± 0
TDS (ppm)	135.7 ± 0	217.2 ± 0	151.2 ± 0	176.4 ± 0	174.9 ± 0	4.395 ± 0
EC (mS/cm)	271 ± 0	448.7 ± 0	372.2 ± 0	353.2 ± 0	349.8 ± 0	558.8 ± 0
Secchi depth (cm)	20.333 ± 0.57	30 ± 0	21 ± 0	20 ± 0	18 ± 0	18 ± 0
pH	7.639 ± 0	7.707 ± 0	8.412 ± 0	8.612 ± 0	8.891 ± 0	7.537 ± 0
Total hardness (ppm)	75 ± 0	75 ± 0	75 ± 0	75 ± 0	75 ± 0	75 ± 0
Total alkalinity (mg/L)	100 ± 0	130 ± 0	100 ± 0	80 ± 0	80 ± 0	120 ± 0
Dissolved nitrate (µM)	73.548 ± 0	50.968 ± 0	60.323 ± 0	71.290 ± 0	80.538± 0.0005	83.978± 0.0005
Dissolved ammonium (µM)	0	0	0	0	0	0
Dissolved *o*-phosphate (µM)	4.34 ± 0.001	8.03 ± 0	5.37 ± 0.0005	6.18 ± 0	6.74 ± 0.001	5.81 ± 0.0005
Reactive silicate (µM)	205.63 ± 0	150.83± 0.0005	154.69 ± 0	162.81 ± 0	83.44 ± 0	96.25 ± 0

**Table 2:** The concentration of metals and metalloids in surface water of DPB1, DPB3 and DPB5 of Deepor Beel, Assam, India.

Metal/Metalloid (ppb)	DPB1	DPB3	DPB5
Na	1,016,000	1,035,000	1,021,000
Mg	4433	4347	4418
P	84.15	228.9	87.12
Ca	17,340	16,510	17,170
Cr	0.529	0.525	0.394
Mn	186.5	280.7	238
Fe	148.3	557.2	98.85
Co	0.358	0.383	0.343
Ni	0.509	0.489	0.389
Cu	1.785	2.224	1.516
Zn	2.289	3.195	2.186
Cd	0.037	0.043	0.032
Pb	0.257	0.283	0.191
As	0	0	0

**Fig. 1:**
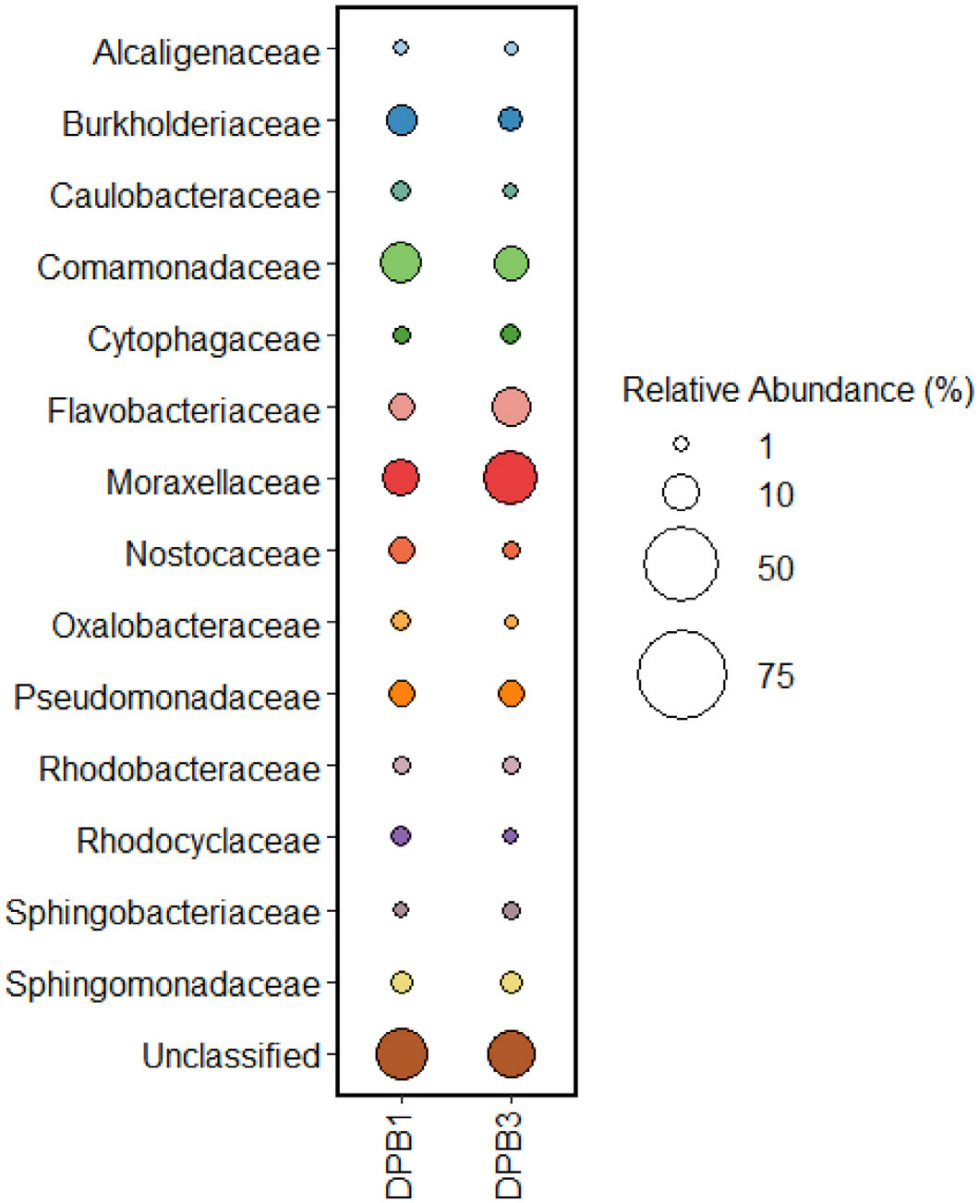
The representation of most abundant bacterioplankton phyla encountered from two stations DPB1 and DPB3 based on the analysis of environmental DNA (eDNA).

Approximately, 237 MB of data was generated from eDNA representing DPB1 and DPB3 stations. The raw sequence data can be accessed at the repository database NCBI with direct URL to the data https://www.ncbi.nlm.nih.gov/sra/PRJNA986129. Dominant bacterioplankton families including Alcaligenaceae, Burkholderiacea, Caulobacteraceae, Comamonadaceae, Cytophagaceae, Flavobacteriaceae, Moraxellaceae, Nostocaceae, Oxalobacteraceae, Pseudomonadaceae, Rhodobacteraceae, Rhodocyclaceae, Sphingobacteriaceae, Sphingomonadaceae, were identified. Among these families, Burkholderiaceae (DPB1–6.4 %, DPB3–3.07 % of the total abundance), Caulobacteraceae (DPB1–1.8 %, DPB3–0.97 % of the total abundance), Comamonadaceae (DPB1–13.2 %, DPB3–7.9 of the total abundance), Flavobacteriaceae (DPB1–4.3 %, DPB3–11.8 % of the total abundance) and Nostocaceae (DPB1- 4.3 %, DPB3- 1.3 % of the total abundance) showed distinct variations in abundance between the two stations. The difference in the relative abundance of these families seen in the two studied stations is shown in Fig. 1.

**Fig. 2:**
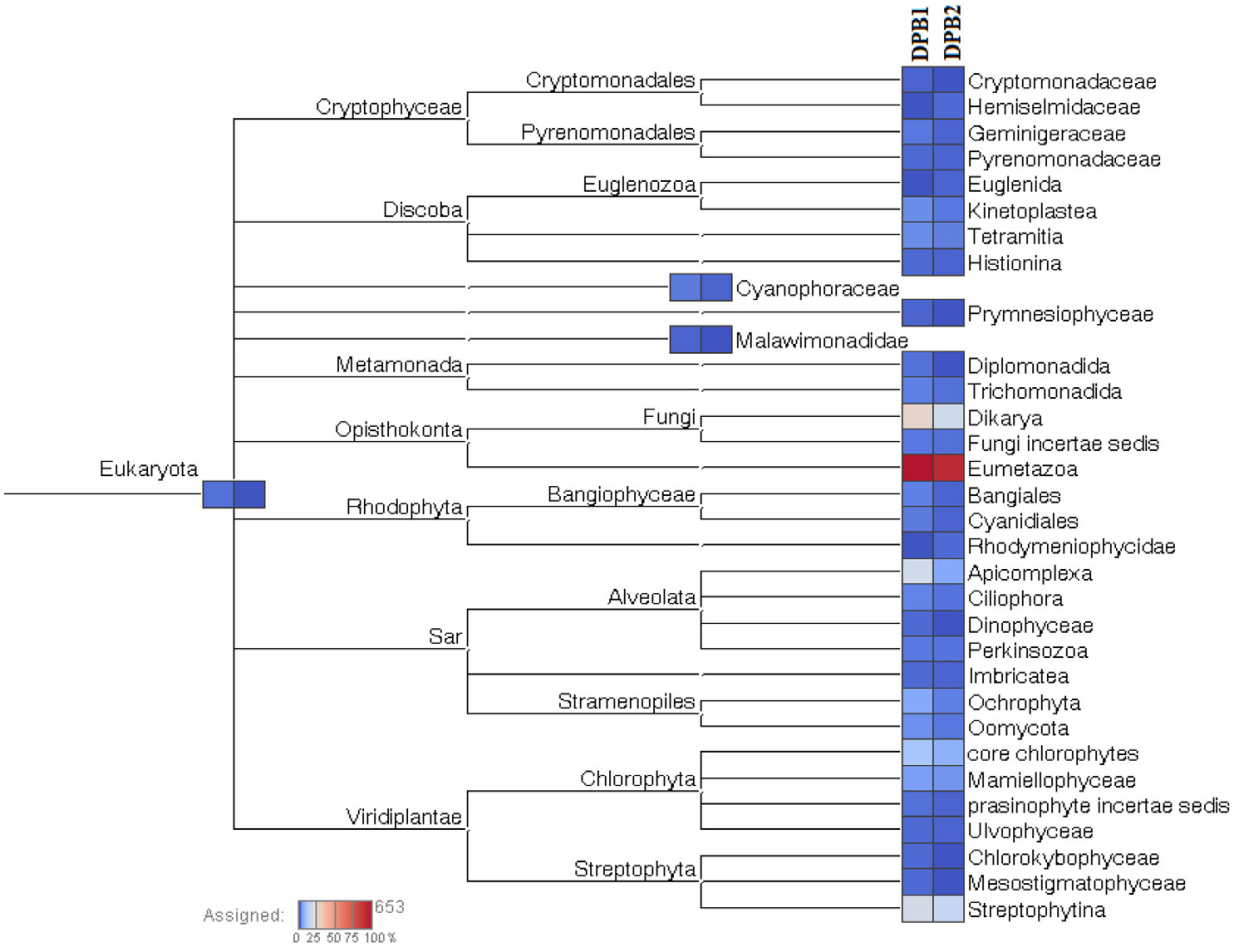
Cladogram showing the relative abundance of identified eukaryotic communities in eDNA representing surface water of DPB1 and DPB3 in Deepor Beel, Assam.

Many eukaryotic affiliations were also seen in the studied datasets, including Chlorophyta, Fungi, Dinophyta and Apicomplexa. There was high abundance of Eumetazoa sequences across the studied stations. The affiliated taxa also showed a difference in abundance between the two studied stations. Differences in abundance was noted for Cercopithecidae (DPB1–1.16 %, DPB3–0.5 % of the total abundance), Didelphidae (DPB1- 4.9 %, DPB3- 2.5 % of the total abundance), Edwardsiidae (DPB1–3.88 %, DPB3–22.6 % of the total abundance), Hydridae (DPB1–6.5 %, DPB3–10.86 % of the total abundance), Onchocercidae (DPB-3.108 %, DPB3–0.94 % of the total abundance) and Volvocaceae (DPB1- 2.79 %, DPB3- 1.68 % of the total abundance) between the two studied stations. Besides, signals of Cyprinidae were detected in both stations, albeit in very low abundance. A cladogram showing the taxonomic affiliations of the eukaryotic communities found in the two studied stations is shown in Fig. 2. The difference in color shows their differential abundance in the two stations.

## 3 Experimental Design, Materials and Methods

### 3.1 Study Site

Deepor Beel, a perennial freshwater lake of approximately 4000 hectares and a RAMSAR Site, has been formed by a former channel of the Brahmaputra River. The wetland is located in the southwest corner of Guwahati City of Assam in India. It is also a Wildlife Sanctuary and home to 200 species of birds including 70 species of migratory birds. In Deepor Beel droppings from avifauna may influence water quality during certain seasons. This wetland is fed by the waters of the Brahmaputra River through a connecting channel known as Khana Jan, which functions both as an inlet and an outlet [7]. The Beel provides a multitude of ecosystem services, encompassing biological diversity, traditional fisheries, tourism, and livelihood support, while also serving as a crucial stormwater retention basin for Guwahati City [1]. Deepor Beel directly sustains the livelihood of more than 800 households through fisheries and the total estimated fishing value is INR 11,64,69,375 per annum [2]. In recent years, anthropogenic stressors have been generated from ongoing activities including construction activities, dumping of mixed solid waste and release of untreated sewage such as from the Pamohi River [3].

### 3.2 Sampling

In the post-monsoon season of 2022 (December), sampling was undertaken in Deepor Beel, Assam (DPB1; 26.114 N 91.661E, DPB2; 26.117 N 91.663E, DPB3; 26.119 N 91.661E, DPB4; 26.117 N 91.658E, DPB5; 26.114 N 91.659E and DPB6; 26.113 N 91.659E). From each station, 1 L of surface water was collected, which was immediately fixed with molecular-grade absolute ethanol (Merck, Germany). The collected water samples were immediately taken to the laboratory for further examination. In addition, 1 L of surface water from each station was also collected and immediately fixed with buffered formalin (4 % final concentration; Merck, Germany). These samples were also used to estimate the total hardness and total alkalinity following standardized methodologies [4], as well as to estimate the concentration of dissolved nutrients.

### 3.3 Measurement of *In*-*Situ* Environmental Parameters

During the time of sampling, *in*-*situ* environmental parameters namely, Air Temperature (AT in °C; digital thermometer, Eurolab, Belgium), Surface Water Temperature (SWT in °C; digital thermometer, Eurolab, Belgium), pH (HI98192, Hanna Instruments Ltd., Romania), Dissolved Oxygen (DO in mg/L; HI98192, Hanna Instruments Ltd., Romania), Electrical Conductivity (EC in µS/cm; HM Digital EC/TDS/TEMP COM-100, Myron L Company, USA), Total Dissolved Solids (TDS in ppm; HM digital EC/TDS/TEMP meter COM-100, Myron L Company USA) and Secchi depth (Secchi disk in cm, LaMotte, France) were measured in triplicates.

### 3.4 Measurement of Dissolved Nutrients

Following standard published protocols, dissolved nitrate, ammonium, *o-*phosphate and reactive silicate concentrations were analyzed based on validated protocols used across diverse freshwater ecosystems [4]. All measurements were done in triplicates using a UV–Vis Spectrophotometer (Hitachi U2900, Japan).

### 3.5 Metal and Metalloid Analyses Using Inductively Coupled Plasma Mass Spectrometry (ICP-MS)

To measure the concentration of metals and metalloids, surface water of DPB1, DPB3 and DPB5 were considered keeping in mind the mixing of pollutants if any, with respect to DPB1 in particular. The collected water was filtered through 0.22 µm, 25 mm nitrocellulose syringe filters (Whatman, United Kingdom) and fixed on-site with Suprapur nitric acid (Merck, Germany). The concentration of metals and metalloids were measured using standard (FINAR-92, Christiansburg, United States of America) and quantified with multi-elemental analyser Inductively Coupled Plasma Mass Spectrometry (ICP-MS), (*XSERIES 2*, Thermo Scientific, USA). During the analysis, BCR 617 and BCR 610 (EVISA, EU) Certified Reference Materials (CRMs) standards were used for calibration. Throughout the analysis, gas pressure was maintained between 120 and 130 pounds per square inch (PSI) with ignition power of 1000 W.

### 3.6 Environmental DNA Extraction (eDNA) and Nanopore Sequencing

Environmental DNA (eDNA) was extracted following published protocol [5]. 200 ng of purified extracted eDNA from each of the stations DPB1 and DPB3 was used to generate the libraries using the Ligation Sequencing Kit (SQK-LSK109, Oxford Nanopore Technologies, United Kingdom) and the native barcoding kit (EXP-PCR096, Oxford Nanopore Technologies, United Kingdom). The adapter and barcode ligated libraries were sequenced for 48-h sequencing cycle on the Nanopore MinION (Oxford Nanopore Technologies, Oxford, United Kingdom) with SpotON Flowcell R9.4 (FLO-MIN106). Guppy v2.3.4 (available from https://community.nanoporetech.com) was used for base-call and demultiplexing the nanopore raw reads (fast5 format) in fastq format.

### 3.7 Raw Data Processing

The raw reads in fastq format were uploaded on MG-RAST [6] and normalized following quality control, which involves removing duplicate reads and trimming the adapters and barcodes. Taxonomic identified was performed using clustering and similarity-based annotation against SILVA v138 database.

## Limitations

Not applicable.

## Ethics Statement

The work outlined above did not involve human or animal subjects; therefore, no regulatory compliance guidelines were applicable.

## CRediT authorship contribution statement

**Rajkumari Nikita:** Investigation, Formal analysis, Data curation, Writing – original draft, Writing – review & editing. **Anwesha Ghosh:** Investigation, Formal analysis, Data curation, Writing – review & editing. **:** Investigation, Writing – review & editing. **Chakresh Kumar:** Investigation, Writing – review & editing. **Arkaprava Mandal:** Investigation, Writing – review & editing. **Nirupama Saini:** Investigation, Formal analysis, Data curation, Writing – review & editing. **Sourabh Kumar Dubey:** Conceptualization, Resources, Writing – review & editing. **Kalpajit Gogoi:** Investigation, Writing – review & editing. **Francois Rajts:** Writing – review & editing. **Ben Belton:** Conceptualization, Resources, Writing – review & editing. **Punyasloke Bhadury:** Conceptualization, Resources, Methodology, Investigation, Writing – review & editing.

## Data Availability

eDNA dataset of Deepor Beel (Original data) (NCBI)
